# Diffuse pulmonary meningotheliomatosis with pan-TRK expression by immunohistochemistry: a novel finding and potential pitfall

**DOI:** 10.1186/s13000-023-01292-1

**Published:** 2023-02-13

**Authors:** Cansu Karakas, Michael A. Nead, Moises J. Velez

**Affiliations:** 1 Department of Pathology & Laboratory Medicine, University of Rochester Medical Center, Rochester, NY USA; 2 Department of Medicine, Pulmonary Diseases and Critical Care, University of Rochester Medical Center, Rochester, NY USA

**Keywords:** Diffuse pulmonary meningotheliomatosis, Meningothelial-like nodule, Lung, Immunohistochemistry, Pan-TRK, *NTRK*

## Abstract

**Background:**

Pulmonary meningothelial-like nodules (PMNs) are benign proliferations of unclear clinical significance. They are mainly asymptomatic lesions that are usually discovered during the pathologic evaluation of resected pulmonary specimens or following post-mortem examination. Diffuse pulmonary meningotheliomatosis (DPM), which presents as bilateral multiple PMNs throughout the lungs, has been described less frequently. DPMs are benign lesions associated with both neoplastic and non-neoplastic pulmonary conditions.

**Case presentation:**

We report the case of a 59-year-old female patient who presented with a history of cough. Computerized tomography (CT) imaging revealed multiple subcentimeter bilateral pulmonary nodules. transbronchial biopsies were obtained which revealed foci of nodular interstitial proliferations composed of epithelioid to spindled cells in a vague whorled pattern. Immunohistochemical stains were diffusely positive for EMA and progesterone receptor. Furthermore, pan-TRK exhibited strong and diffuse membranous expression in the lesional cells. INSM1 was negative for expression. RNA-based next-generation sequencing for the detection of *NTRK* fusions was performed and was negative for gene rearrangements involving *NTRK1*, *NTRK2*, and *NTRK3*.

**Conclusion:**

Here, we report a rare case of DPM and report pan-TRK expression in PMNs which has not been described. We provide a brief review of the literature and provide insight into the potential physiologic nature of PMNs. Lastly, we emphasize the recognition of pan-TRK immunoexpression in PMNs to avoid potential diagnostic errors.

## Introduction

Pulmonary meningothelial-like nodules (PMNs) are benign lesions composed of small nests of epithelioid cells located within the interstitium of the lungs. The lesions are most often single or multifocal and are typically found incidentally on examination of routine hematoxylin and eosin (H&E)-stained slides. The significance or etiologic origin of PMNs remains relatively unknown, although they are often identified in lung resections performed for neoplastic or interstitial lung disease. PMNs included in the differential diagnosis from a radiographic study are rare, as is the sampling of PMNs from a lung tissue biopsy. An exceedingly rare form of PMNs is characterized by numerous disseminated bilateral pulmonary nodules termed as “diffuse pulmonary meningotheliomatosis (DPM)”. In contrast to PMNs, DPMs may be radiologically interpreted as diffuse interstitial lung disease with diffuse reticulonodular infiltrates and ground glass nodules [1, 2]. Their clinical significance is unclear, with few case reports in the literature. Neurotrophic tropomyosin-receptor kinase (*NTRK*) signaling is orchestrated by neurotrophins and is involved in neural system development [3]. Tumor-agnostic fusions involving *NTRK1*, *NTRK2* and *NTRK3* are well-known drivers of tumorigenesis in numerous adult and pediatric tumors [4–7]. While immunohistochemistry (pan-TRK) can be reliably used to screen for *NTRK1-3* gene fusions by identifying protein expression, wild-type or physiologic expression has been identified in neural, smooth muscle, and neuroendocrine tissue [8]. Here, we present a rare case of DPM with pan-TRK immunohistochemical expression, which may provide a notable clue to the function of PMNs and provide a brief review of the literature.

## Case presentation

A 59-year-old woman presented to the clinic with chronic cough. Multiple bilateral lung nodules were observed following computed tomography (CT) imaging of the chest and abdomen, which was initially performed to investigate irritable bowel disease. Her medical history included type-2 diabetes mellitus, gastroesophageal reflux disease, deep vein thrombosis, depression, and anxiety. She was a nonsmoker with no significant history of malignancy. Clinically, the patient reported night sweats, fatigue, productive cough, and occasional shortness of breath. Initial physical examination and laboratory test results were unremarkable. Her spirometry results were within normal ranges. An extensive laboratory workup was negative for autoimmune and infectious etiologies. The patient underwent transbronchial biopsies with multiple samples taken from the left lung to confirm the diagnosis. Follow-up imaging showed no progression in nodule size or number.

### Imaging findings

**Fig. 1:**
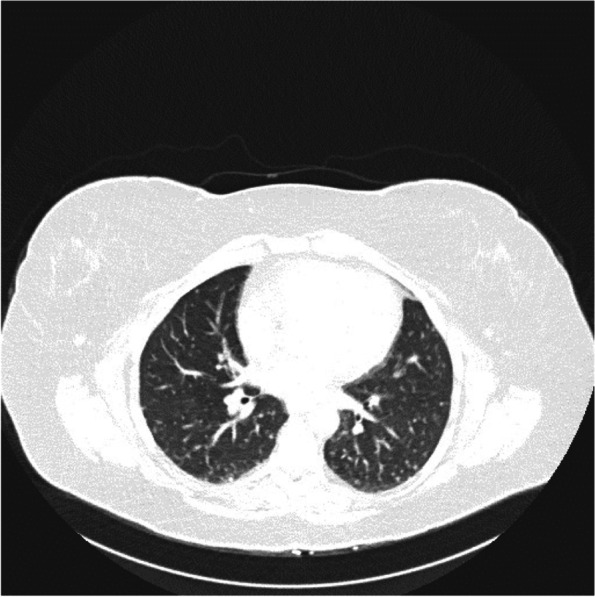
Imaging findings. Selected axial non-contrast chest CT image shows bilateral multiple small nodules randomly distributed through both lungs

Chest CT revealed numerous bilateral pulmonary nodules throughout the lungs, most of which were small in size, measuring up to 5 mm in the right lower lobe and up to 4 mm in the left upper lobe (Fig. 1).

### Pathologic findings

**Fig. 2:**
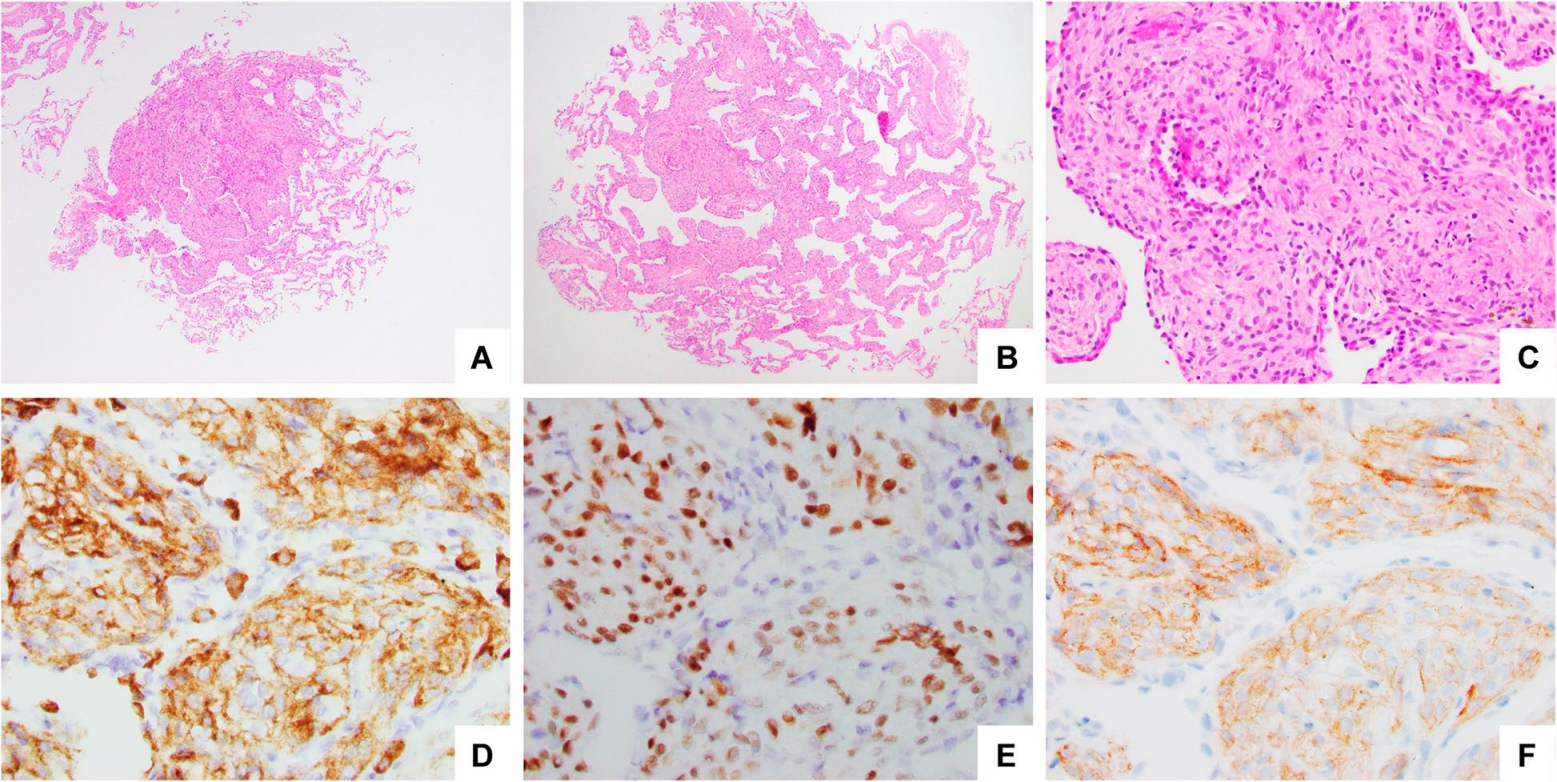
Histological and immunohistological findings. **A-B** H&E sections showed meningothelial-like nodules which are distinct from the neighboring normal lung architecture. **C** Higher magnification shows an interstitial proliferation of epithelioid and spindled cells with a vague whirling and streaming pattern. **D** The cells comprising the nodules showed diffuse cytoplasmic staining with EMA (**E**), strong nuclear PR expression (**E**) and diffuse membranous expression with pan-TRK (**F**). Original magnification: x100 (A-B), x200 (**C-F**).

H&E-stained sections show nodular foci of interstitial epithelioid to spindled cells with eosinophilic cytoplasm and oval nuclei with fine chromatin arranged in a vaguely whorled architecture (Fig. 2A-C). Immunohistochemical evaluation of the lesional cells showed positive expression for EMA (membranous expression), progesterone receptor (nuclear expression), and pan-TRK clone mAb EPR17341 (membranous expression). Pan-TRK expression was restricted to the lesional cells and was not present in the background lung parenchyma. Insulinoma-associated protein 1 (INSM1) was negative for expression (Fig. 2D-F). Subsequent biopsies targeting nodules of the contralateral lung showed histologically unremarkable benign alveolated lung parenchyma and was deemed non-diagnostic, owing to the limited targeting ability afforded by bronchoscopic forceps biopsies. RNA-based next-generation sequencing for the detection of *NTRK* fusions was negative for gene rearrangements involving *NTRK1*, *NTRK2*, and *NTRK3*.

## Discussion & conclusion

PMNs are rare lesions that are most commonly observed incidentally, with a rate of 0.07–13.8% during the examination of autopsy or surgical lung specimens [2, 9–11]. They primarily occur in middle-aged women and frequently in the sixth and seventh decades of life [11, 12]. This entity was first defined by Korn et al. in 1960 as pulmonary lesions associated with small veins, and they proposed the term “minute pulmonary chemodectomas” based on cytologic features, cellular organization, and its relationship with vessels [12]. In 1988, Gaffey et al. proposed the term “minute pulmonary meningothelial-like nodules” (MPMNs) after investigating the origin of these lesions with an extensive immunohistochemistry work-up and demonstrating the meningothelial nature with strong expression of vimentin and EMA, which are two markers associated with cranial and extracranial meningiomas [9]. Of note, the PMNs revealed no expression of cytokeratins, S-100 protein, neuron-specific enolase, or actin [9]. Other subsequent studies reinforced these findings by showing that PMNs and meningiomas have identical cytological and immunohistochemical features [10, 11, 13, 14]. Ultrastructural studies of these lesions have also supported their meningothelial nature by demonstrating cells with cytoplasmic interdigitating junctions, desmosomes and intracytoplasmic filaments [9, 15]. Some authors have reported PMNs with myosin expression, suggesting a myogenic origin [16]. It has also been suggested that sex steroid hormones may play a role in their development, as evidenced by a stronger female preponderance compared to men [17]. However, although a female predominance has been reported, progesterone expression in PMNs in males suggests that the receptor status is independent of hormonal status and race [18]. In the present case, the PMNs were positive for EMA, PR, and pan-TRK, but negative for INSM1, a marker of neuroendocrine differentiation. The pathologic, radiologic, and clinical findings supported the diagnosis of pulmonary meningotheliomatosis.

Multiple PMNs (MPMNs), alternatively termed “Diffuse pulmonary meningotheliomatosis” (DPM), is exceedingly rare with few reports in the literature [1, 2, 19–24]. In the original report by Korn et al., one patient presented with multiple lesions involving all lobes [12]. In most cases, multiple lesions are localized or confined to one side of the lung rather than showing diffuse bilateral involvement [1, 11, 19]. Our patient had numerous nodules in both lungs, making our case unique. The radiographic features of DPM include numerous nodules (100 μm to 3 mm) randomly distributed throughout the lung interstitium, some of which may have central cavitation [2, 9, 19, 20, 25]. Diffuse bilateral involvement of the lung parenchyma can clinically suggest a variety of interstitial pulmonary processes, including idiopathic interstitial pneumonia and lymphangitis carcinomatosa [26]. Furthermore, DPM can simulate miliary metastatic disease, particularly in patients with a history of malignancy, and may cause misstaging [27]. Transbronchial or surgical (VATS or open) biopsies can be used to confirm the diagnosis. Similarly, numerous bilateral pulmonary nodules throughout the lungs in our case were suspected to be metastatic, infectious, or granulomatous. Based on these clinical symptoms and radiologic findings, our patient underwent transbronchial biopsies with samples drawn from both lungs to help confirm the diagnosis.

The etiology of this disease remains unclear. The clinical conditions most commonly associated with PMNs include thromboembolic disease, respiratory bronchiolitis-associated interstitial lung disease, desquamative interstitial pneumonia, and atypical adenomatous hyperplasia of the lungs [11, 12, 15, 28, 29]. The simultaneous occurrence of lesions in patients with PMNs and organizing pulmonary thromboemboli suggests that they may be acquired secondary to ischemia resulting from vascular occlusion [11]. The absence of PMNs in pediatric lung specimens indicates that they are not congenital [11]. The low incidence of these lesions in acute lung injury suggests that they are associated with chronic conditions rather than acute lung injury [11]. Mizutani examined 1724 patients who underwent lung resection and identified 271 MPMNs in 121 patients. The incidence of MPMNs was significantly higher in malignant pulmonary tumors than in benign diseases (*p* = 0.044). Furthermore, the prevalence of MPMNs was significantly higher in patients with adenocarcinoma than in those with other primary lung cancers (*p* < 0.01) [28]. Gleason et al. reviewed the literature through 2016 for reported DPM cases and showed that 44% of the patients had a history of active malignancy at the time of their DPM diagnosis. Only 12% of the patients were diagnosed by transbronchial biopsy, while the remainder underwent surgical lung biopsies [22]. Our patient was a middle-aged woman with non-specific pulmonary symptoms. After extensive work-up, no underlying neoplastic or non-neoplastic pulmonary diseases were detected, and the diagnosis of PMNs was confirmed following transbronchial biopsies.

There are very few molecular studies on the pathogenesis of this disease. Immunohistochemical and clonal analysis of these lesions by Niho et al. showed partial monoclonal expansion in seven out of 11 lesions using the X-chromosome-linked human androgen receptor gene assay (HUMARA) [10]. In this study, the authors did not find any histologic differences between monoclonal and polyclonal meningothelial nodules, and thus concluded that since only a portion of these lesions showed clonal expansion, they most likely represent a reactive process rather than a clonal neoplastic process [10]. More recently, Ionescu et al. compared 33 minute pulmonary meningothelial-like nodules (MPMNs) and 10 benign meningiomas using IHC and mutational analysis in 16 cases [30]. Reasons for surgical resection included carcinoid tumors, hypersensitivity pneumonitis, and metastatic carcinoma. A minimum of four pulmonary MPMN nodules were identified by CT scan and confirmed and classified by microscopic examination as “MPMN-omatosis syndrome” [30]. The authors found that the highest loss of heterozygosity was observed on chromosomes 22q, 14q, and 1p in meningiomas not shared by MPMNs. All solitary MPMN had < 1 LOH events compared to greater genetic stability. Conversely, multiple LOHs in various chromosomal loci triggering the inactivation of tumor suppressor genes, including VHL, p16, p53, and *NF2*, have been observed in MPMN-omatosis syndrome. The authors concluded that the two processes (meningioma and MPMNs) are unrelated, and when the lesions are multiple and diffuse, they might represent a transition between a reactive and neoplastic proliferation [30]. Another recent study on the characterization of MPMNs as precursor neoplastic lesions compared MPMNs (*n* = 2) and CNS meningiomas (*n* = 2) using fluorescent in situ hybridization (FISH) and found two deletions of the *NEF-2* gene in two MPMNs and one deletion of *NF-2* in two CNS meningiomas, suggesting that they are related lesions and may originate from the same precursor cells [31]. Other studies demonstrated similar findings using FISH analysis and provided support for the hypothesis that MPMNs and pulmonary meningiomas have common genetic pathways and may arise from the same precursor cells [32].

The differential diagnoses of diffuse MPMNs include metastatic carcinomas, pulmonary meningiomas, and pulmonary carcinoid tumorlets. The widespread distribution of the lesions throughout the lung can be misdiagnosed as metastatic disease. Pulmonary meningiomas are clonal neoplastic lesions which usually present as mass lesions rather than minute nodules [33]. Spinelli et al. suggested that pulmonary meningiomas may arise from meningothelial-like nodules, however, the simultaneous presence of meningothelial-like nodules and meningioma is exceedingly rare [13]. Pulmonary carcinoid ‘tumorlets’ are defined as groups of neuroendocrine cells measuring less than 0.5 cm; usually located in association with an airway and are often confused with meningothelial nodules [34]. Cytologically, tumorlets consist of uniform cells with oval, round or spindle shaped nuclei with “salt and pepper” or speckled chromatin; however, PMN cells display a more epithelioid appearance with nests of oval, round cells with eosinophilic cytoplasm and indistinct cell borders that may contain intranuclear cytoplasmic inclusions [14]. If it is cytologically difficult to discern a carcinoid tumorlet from PMNs, weak cytoplasmic reactivity for cytokeratin and positive expression of chromogranin, synaptophysin, or INSM1 in carcinoid tumorlets is helpful in distinguishing carcinoid tumorlets from PMNs [35, 36]. Similarly, our patient expressed EMA and PR, while it was negative for INSM1, which supported our diagnosis. PMNs may be distinguished histologically by small syncytial whorls of bland epithelioid to spindle cells with pale eosinophilic cytoplasm located in the interstitial space or surrounding small veins; any degree of cellular atypia, particularly in cytology specimens, should be a warning for malignancy [18, 37]. In such a scenario, IHC would be helpful in ruling out the possibility of carcinoma [34].

NTRKs are a family of transmembrane tyrosine kinases that are expressed in neural tissues and play a key role in the development and function of the neural system [38–40]. Three members of this family, TRKA, TRKB, and TRKC, are encoded by the proto-oncogenes, *NTRK1*, *NTRK2*, and *NTRK3*, respectively [41, 42].

Gene fusions involving *NTRK* genes are known to have oncogenic potential. Oncogenic fusions between the C-terminal kinase domain and the N-terminal fusion partner of the *NTRK* genes have been identified in high prevalence in rare subtypes of pediatric and adult tumors, such as secretory carcinoma of the breast, secretory carcinoma of the salivary gland, and infantile fibrosarcoma, while implicated in a small percentage of common cancers arising in the adult population [6, 7, 43]. Our prior knowledge of pan-TRK expression in PMNs was discovered during our institutional validation of the Ventana clone EPR17341 assay as a screening modality for actionable *NTRK* fusions. Given our knowledge of pan-TRK expression in PMNs, we sought to explore the possibility of *NTRK* fusions as an oncogenic driver in the current case of DPM.

The pan-TRK rabbit monoclonal antibody reacts with the C-terminus of TRKA, TRKB, and TRKC proteins, which are known to be conserved across wild-type and chimeric fusion proteins [44]. The pan-TRK antibody binds to the C-terminus or 3’ end of NTRK in tissues normally expressing NTRK and binds to the 3’ end of *NTRK* fusions, which are conserved among *NTRK* gene rearrangements [8, 44]. As evidenced in this case, pan-TRK protein expression was identified in PMNs; however, next-generation sequencing for *NTRK* gene rearrangements involving *NTRK1*, *NTRK2* and *NTRK3* showed negative results. Hence, we confirmed the absence of detectable *NTRK* fusion in PMNs using an RNA-based next-generation sequencing assay and confirmed an adequate sample of PMNs for sequencing analysis. Given these findings, we conclude *NTRK* fusions are not implicated in DPM, and that PMNs likely show wild-type or physiologic NTRK expression.

Interestingly, gene expression analysis of 68 cranial meningiomas by Lee et al. revealed multiple genes involved in kinase signaling pathways, including a subset of meningiomas expressing *MYLK, PRKD1, NTRK2, ROR1, TNIK, and PRKG1* [45]. Although *NTRK* fusions have been described in various CNS brain tumors [46], *NTRK* fusions involving cranial meningiomas have not been implicated in tumorigenesis. Based on the gene expression profile of meningiomas, we may expect to see wild-type or physiologic pan-TRK IHC expression, as evidenced in PMNs, supporting regulation by neurotrophins. Given the similarities between PMNs and meningiomas, both morphologically and immunohistochemically, RNA transcription analysis of PMNs may provide further insight into the various signaling pathways expressed in PMNs, possibly providing further insight into the precise function (if any) and etiology of these enigmatic lesions. Furthermore, wild-type expression of pan-TRK in PMNs should be recognized to avoid misinterpretation of pan-TRK results in lung neoplasms where PMNs may be entrapped by tumor or arise adjacent to the tumor bed.

## Data Availability

All data supporting the findings and conclusions of this case report is included within the article.

## Abbreviations

PMNs: Pulmonary meningothelial-like nodules
DPM: Diffuse pulmonary meningotheliomatosis
NTRK: Neurotrophin tropomyosin-receptor kinase
CT: Computed tomography
MPMNs: Minute pulmonary meningothelial-like nodules
